# Molecular Flexibility of Antibodies Preserved Even in the Dense Phase after Macroscopic Phase Separation

**DOI:** 10.1021/acs.molpharmaceut.1c00555

**Published:** 2021-10-12

**Authors:** Anita Girelli, Christian Beck, Famke Bäuerle, Olga Matsarskaia, Ralph Maier, Fajun Zhang, Baohu Wu, Christian Lang, Orsolya Czakkel, Tilo Seydel, Frank Schreiber, Felix Roosen-Runge

**Affiliations:** † Institut für Angewandte Physik, Universität Tübingen, Auf der Morgenstelle 10, 72076 Tübingen, Germany; ‡ Institut Laue-Langevin, 71 Avenue des Martyrs, 38042 Grenoble, France; § Jülich Centre for Neutron Science JCNS at MLZ, Forschungszentrum Jülich, Lichtenbergstraße 1, 85748 Garching, Germany; ∥ Department of Biomedical Science and Biofilms-Research Center for Biointerfaces (BRCB), Malmö University, 205 06 Malmö, Sweden

**Keywords:** antibody therapy, molecular flexibility, dynamics, diffusion

## Abstract

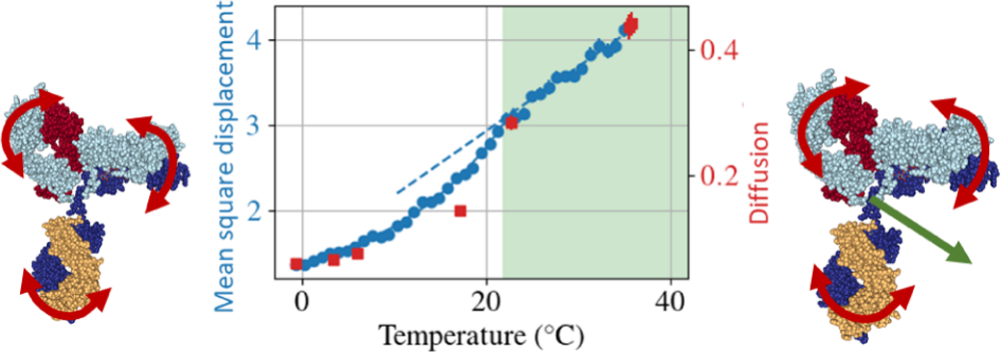

Antibody therapies are typically based on high-concentration formulations that need to be administered subcutaneously. These conditions induce several challenges, inter alia a viscosity suitable for injection, sufficient solution stability, and preservation of molecular function. To obtain systematic insights into the molecular factors, we study the dynamics on the molecular level under strongly varying solution conditions. In particular, we use solutions of antibodies with poly(ethylene glycol), in which simple cooling from room temperature to freezing temperatures induces a transition from a well-dispersed solution into a phase-separated and macroscopically arrested system. Using quasi-elastic neutron scattering during *in situ* cooling ramps and in prethermalized measurements, we observe a strong decrease in antibody diffusion, while internal flexibility persists to a significant degree, thus ensuring the movement necessary for the preservation of molecular function. These results are relevant for a more dynamic understanding of antibodies in high-concentration formulations, which affects the formation of transient clusters governing the solution viscosity.

## 1 Introduction

High-concentration liquid formulations (HCLFs) of antibodies typically have concentrations larger than 50 mg/mL, which are required to allow a simple and fast admission of a sufficient antibody dose for medical treatments in subcutaneous delivery.^1−3^

These formulations face different challenges that limit the possible applications of HCLFs, which are linked to their phase diagram: antibody solutions at high concentrations can show a strongly elevated viscosity,^4^ which prohibits injection. A better understanding of molecular determinants of viscosity in antibody formulations is highly desired. Furthermore, the presence of a liquid–liquid phase separation (LLPS) region, in which the solution phase-separates into two liquid phases,^5−8^ and aggregation,^9,10^ limits macroscopic sample stability. Finally, it is essential to ensure that macromolecular crowding does not impact the molecular properties in an irreversible way.

Colloid theory and, in general, soft matter approaches, have been fruitful in providing a conceptual picture to estimate the rheological and aggregation properties of HCLF.^4,7,11−23^ In these studies, the central objective has been to predict the macroscopic properties of antibody solution such as viscosity from molecular interactions, in order to avoid undesired sample properties.

A second strand of research into well-dispersed sample states focuses on the microscopic molecular dynamics, which is linked to their interactions, as a dynamical cornerstone to understand the macroscopic density relaxations. The investigation of the molecular dynamics of antibodies showed the possibility to model the movements of proteins and their domains and to give information about cluster formation.^14,20,24−30^ Preservation of molecular flexibility is an essential ingredient to effectively functioning proteins, and these studies suggested overall that molecular flexibility is preserved in well-dispersed and stable states. Little is known, though, to which extent internal flexibility is affected by solution states occurring at high concentrations typical of HCLF.

In this study, we investigate an antibody system which shows both LLPS and strongly elevated protein concentrations close to an arrest transition. The solution is composed of a bovine polyclonal antibody, γ-globulin, and a polymer, polyethylene glycol (PEG). The presence of PEG increases the attraction between the proteins through depletion interaction, giving rise to phase separation at room temperature.^31^ The sample was probed in a temperature range covering both the stable one-phase region and phase-separated states after LLPS, allowing for a comparison of the molecular dynamics in the two states. At low temperature, the emerging dense phase locally reaches a concentration so elevated that the phase separation kinetics is strongly slowed down.^31−33^ Using two types of neutron spectrometers probing quasi-elastic scattering, we establish an access to the diffusive dynamics in HCLF on length scales from several protein–protein distances down to the protein side chain distances and on timescales from several tens of picoseconds to hundreds of nanoseconds. We probe the effects of temperature quenches into the LLPS region on antibody diffusion as well as domain and internal dynamics of the antibodies.

## 2 Materials and Methods

### 2.1 Sample Preparation

The sample preparation of the parent solution followed an established protocol,^31^ modifying it to the needs of the D_2_O solvent for neutron spectroscopy. A parent solution of 100 mg/mL bovine polyclonal γ-globulin (Sigma-Aldrich, SRE0011) and PEG (molecular weight, 1 kDa) was prepared at 6% (PEG weight/volume of the solvent %) in a D_2_O buffer solution with 150 mM NaCl, 20 mM 4-(2-hydroxyethyl)-1-piperazine-ethanesulfonic acid (HEPES) at pH = 7.0 and 2 mM NaN_3_ to prevent bacterial growth. We note that γ-globulin and PEG were not deuterated. This sample was unstable right after preparation, showing high turbidity. It was kept at 21 °C to let the phase separation proceed until a macroscopically visible phase separation was observed. The sample was briefly centrifuged at 6500 rpm to increase the sharpness of the meniscus. After circa 30 min of centrifugation, both the dilute and dense phases were transparent to the eye. The dense phase was extracted and used as sample for all subsequent measurements.

### 2.2 Quasi-Elastic Neutron Backscattering Spectroscopy

Quasi-elastic neutron backscattering (NBS) spectroscopy offers access to molecular dynamics on time and length scales corresponding to the movements of individual proteins and their domains and side chains.

The experiments were performed at the instrument IN16B at Institut Laue-Langevin (ILL), Grenoble, France, which offers an excellent energy resolution of 0.8 μeV, allowing to study motions from roughly 100 ps to 10 ns by investigating an energy transfer range of Δ*E*_max_ = 30 μeV.^34^ IN16B was used with Si(111) monochromator and analyzer crystals, corresponding to an elastic wavelength of 6.27 Å. A linear motor Doppler drive carrying the monochromator was used to define the energy transfer.

The samples were filled into double-walled cylindrical aluminum cans with a 0.15 mm gap and a 23 mm outer diameter, sealed with indium wire, and mounted in a standard orange cryofurnace for temperature control during the data acquisition. The typical duration of one full spectra measurement was 1 h and 20 min. The data were reduced using Mantid^35^ and further analyzed using own python scripts. The data are available in refs (36) and (37).

As the central quantity, the dynamic structure factor *S*(*q*,ω) is measured as a function of the energy transferred ℏω and the scattering vector *q* to investigate the short time diffusion of the system, giving information on the type of the diffusion and on parameters such as the diffusion coefficient or the mean-square displacement (MSD) of a particle.^24,38^ Given the *q* range investigated, the scattering signal obtained at the NBS experiment is mainly dominated by incoherent scattering and thus focus on the short-time self-diffusion. Importantly, the use of D_2_O as the solvent implies that the hydrogen atoms in the protein and in PEG contribute more strongly to the neutron scattering signal under these conditions, and the observed dynamical signatures can thus be unambiguously assigned to the protein.

The analysis performed follows the procedure established in the literature.^39^ The signal is a sum of different contributions, namely the diffusion of the water molecules, the PEG polymers, and the antibodies. The latter is divided in two contributions: one corresponds to the apparent global diffusion of the protein, and the second one to the internal diffusion, as validated in a previous study on the pure antibody solution without PEG,^24^ where the global diffusion corresponds to the rotation and translation of a single antibody, while the internal diffusion corresponds to the movement of the hydrogen atoms in the peptide chain. At temperatures below 21 °C, the sample presents two phases, but only the dense phase contributes significantly to the signal; hence, it is not necessary to add further contributions. The different contributions of the dilute phase and the dense phase are shown in Supporting Information Figure S3a. Considering the estimated volume of the dense phase of 70%, the concentration estimation of 270 mg/mL in the dense phase and 30 mg/mL in the dilute phase,^31^ the molecules in the dilute phase constitute only roughly 5% of the molecules in solution. Each contribution corresponds to a Lorentzian function *L*_γ_(ω) with width γ. Therefore, the dynamic structure factor is modeled by
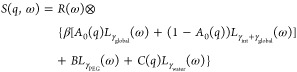
1where γ_global_, γ_internal_, γ_PEG_, and γ_water_ provide information on the relaxation rates of the corresponding component. The so-called elastic incoherent structure factor (EISF) *A*_0_(*q*) characterizes the confined geometry of local internal motions, and *B* and *C*(*q*) denote the amplitudes of PEG and water contributions (see for details below).

The term *R*(ω) represents the resolution function of the instrument, which can be described by a Gaussian function. The measured signal is the convolution (⊗) of the sample signal and the resolution function. *R*(ω) was fitted from the signal of a measurement of vanadium.^40^

**Figure 1:**
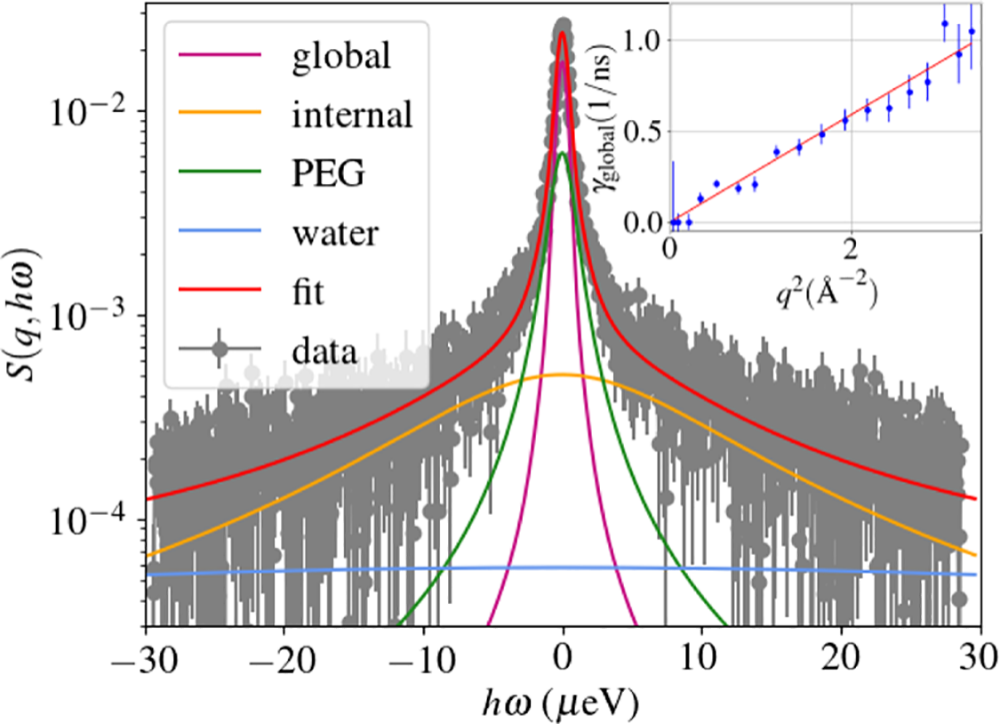
Example of the fitted backscattering data, with the four contributions indicated in the legend. The inset shows the *q*^2^ dependency of the width of the Lorentzian of the global diffusion.

To increase the stability and robustness of the fit, and to include prior knowledge on the system, the *q* dependency of the Lorentzian functions was fixed. The global diffusion and the diffusion of PEG were set to Fickian diffusion (*i.e.,* γ_global_(*q*) = *D*_global_*q*^2^ and γ_PEG_(*q*) = *D*_PEG_*q*^2^), and the internal diffusion was modeled with the jump diffusion signature γ_int_(*q*) = *D*_int_*q*^2^/(1 + *q*^2^*D*_int_τ).^39^ This assumption was validated by the trends obtained from *q*-wise fits (Figures 1 and S4 in the Supporting Information). Because of the limited ω range measured, the values of the width of the Lorentzian function of water were not fitted; instead, tabulated values were used.^40^ A pure D_2_O solution was measured to estimate the amplitude *C*(*q*) of the Lorentzian function of water. The parameter *C*(*q*) was calculated by taking into account the reduction of the volume fraction of water (0.7) due to the presence of protein and polymer. The value of *B* was fitted assuming no *q* dependence to increase the stability of the fit. The value of *A*_0_(*q*) was fit without any prior assumption on the *q* dependence.

*A*_0_(*q*) corresponds to the so-called EISF, and, assuming a localized movement in a radial harmonic potential, it can in a basic model be described with a Gaussian profile (eq 2).^41^
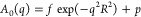
2where *R* denotes the length of the localized motion, *p* is the fraction of immobile hydrogen atoms, and *f* is proportional to the fraction of hydrogen atoms which perform the confined motion.

### 2.3 Elastic Fixed-Window Scans of NBS

**Figure 2:**
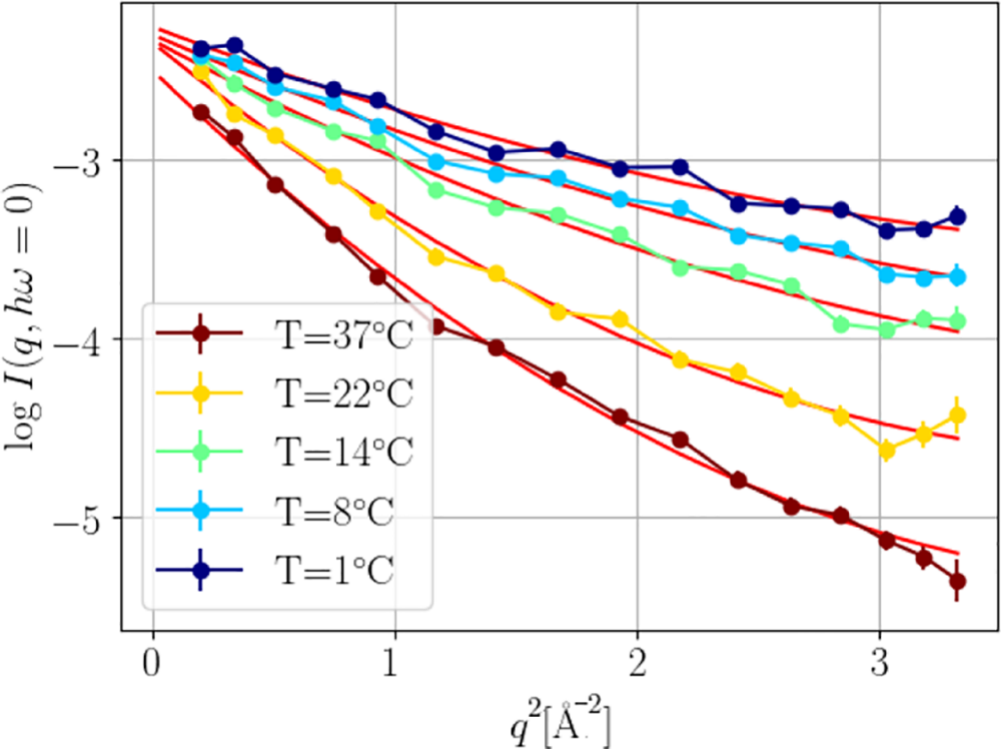
Intensity of the FWS as a function of scattering vector. The respective fits (eq 3) are plotted in red.

The system was monitored during the temperature quench with the so-called elastic fixed window scans (FWSs) which fix the energy transfer to *h*ω = 0 μeV and thus allow monitoring of the dynamical evolution with a higher sampling frequency. In this way, the acquisition time was reduced to 10 s. From the intensity at zero energy transfer, it is possible to obtain information about the apparent (MSD) ⟨*u*^2^⟩ *via* the quadratic function
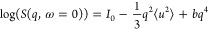
3where *b* is an additional scalar fit parameter.^42^ Typical curves with quadratic fits collected during a quench from 37 to 1 °C are shown in Figure 2.

### 2.4 Neutron Spin-Echo Spectroscopy

Neutron spin-echo (NSE) spectroscopy allows to access short-time diffusion of proteins on length scales of few neighbor shells and timescales of up to several hundreds of nanoseconds and is thus of high relevance to understand how proteins move as a whole in dense solutions.

The measurements were performed at the instrument IN15 at Institut Laue-Langevin, Grenoble, France. The incident wavelength of the neutron beam was 10 Å, resulting in a Fourier time range covered up to 200 ns. The resolution functions of the instruments were determined for each experimental setup using the elastic scattering of graphite. The resulting intermediate scattering functions (ISFs) were corrected for the background dynamics of the buffer solution with PEG at a concentration of 2.5%, which is the estimated concentration of PEG in the dense phase.^31^ Samples were filled into Hellma quartz cuvettes with a 2 mm gap and sealed with parafilm to avoid evaporation. The data are available in ref (37).

The NSE method measures the energy transfer of neutrons at an extremely high energy resolution as a phase shift in the Larmor precession of the neutron spins in a magnetic field.^26^ The *I*(*q*,*t*) curves obtained for the samples were normalized by the signal (*I*(*q*,0)) of a fully elastic scatterer.

From the ISF *I*(*q*,*t*) as the measurement observable, the diffusion coefficient can be determined to evaluate the mobility of the particles in the system. The ISF cannot be fitted by a single exponential decay, as was already seen for the pure antibody solution.^25,29^ The complex composition of the solution and the limited time resolution of the measurement do not allow for a robust fit and a meaningful analysis of all possible decays. Thus, to extract robust characteristics of the dynamics, we used a stretched exponential decay, also called Kohlrausch Williams Watts function^43^
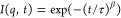
4

To avoid any coupling between the decorrelation time τ and the exponent β, β was fixed to a value of 0.5, which was in the range of preliminary free fits.

The physical interpretation of a stretched exponential involves a distribution of decay processes, with the average decorrelation time ⟨τ⟩ = τ/βΓ(1/β),^44^ with Γ(*x*) being the gamma function. The diffusion coefficient was calculated as *D*_NSE_ = 1/(⟨τ⟩*q*^2^).

### 2.5 Measurement Protocol for Temperature Quenches

Before each temperature quench, the sample was equilibrated for 10 min at 37 °C in the measurement position. Temperature quenches were performed by cooling in the measurement position to the target temperature, avoiding carefully any overshooting of the temperature. The system was followed with elastic FWS in the case of NBS and measuring only one Fourier time for NSE, which allowed us not only to monitor the changes but also to ensure proper equilibrium for the subsequent full measurements at lower temperature. After 1 h, the system was considered to be in equilibrium, and full correlation functions were measured.

## 3 Results and Discussion

Proteins perform a complex set of motions on nanoscopic time and length scales.^45^ The entire protein molecule diffuses in the cage of the neighboring molecules on short timescales, both with translational and rotational displacements. For Y-shaped antibodies, the domain dynamics, that is, the relative motions of the lobes, present a second significant motif. Finally, both the protein backbone and the side chains exhibit locally confined dynamics.

Employing both NBS and NSE with their different characteristics, we aim to obtain information on these three hierarchical levels of dynamics. While a complete decoupling into individual contributions is impossible, we focus on trends with lowering temperature. In particular, NSE mainly measures collective dynamics, at *q* values corresponding to the length scales of nearest neighbors. These scales thus focus on the diffusion of the entire molecule, that is, translational and rotational diffusion, with potentially some contributions from domain dynamics. The larger *q* values probed by NBS allow to also address local motions and monitor the translational and rotational self-diffusion of lobes as well as of the entire protein, depending on the conditions.

### 3.1 Significant Reduction of Diffusive Motions upon Cooling into the Phase-Separated Regime

**Figure 3:**
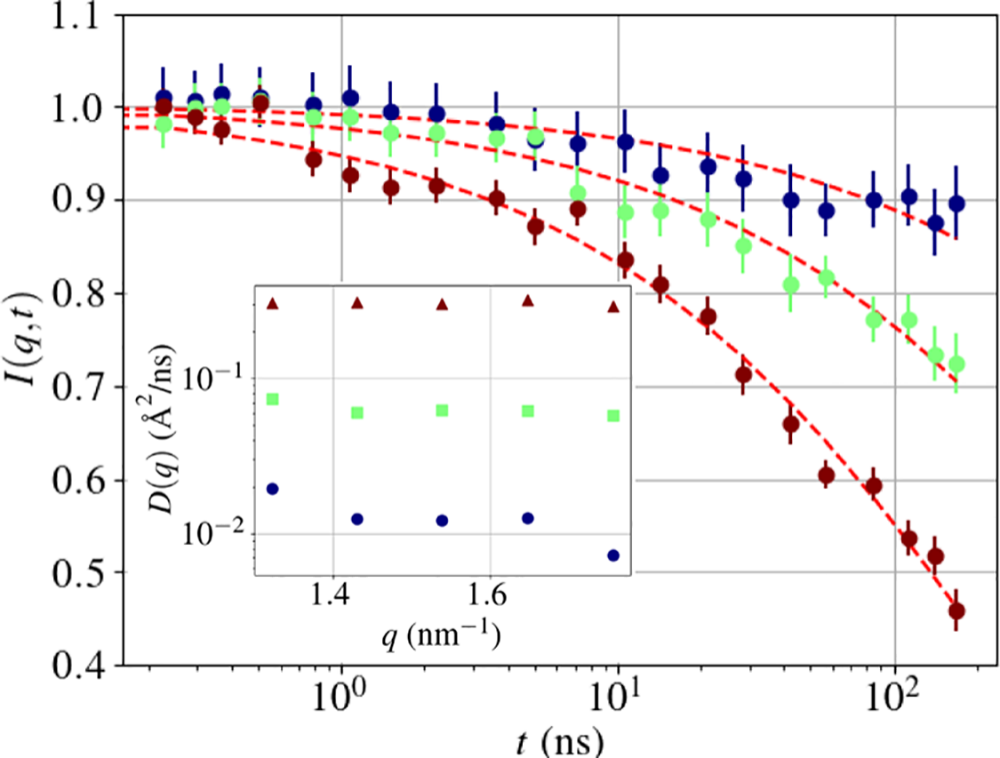
Intermediate scattering function at *q* = 1.54 nm^–1^ measured at 6 °C (blue), 18 °C (green), and 37 °C (red). In the inset, the diffusion coefficient calculated with *D*_NSE_ = 1/(⟨τ⟩*q*^2^) as a function of scattering vector is shown. We remark that 1 Å^2^/ns corresponds to 10^–7^ cm^2^/s.

**Figure 4:**
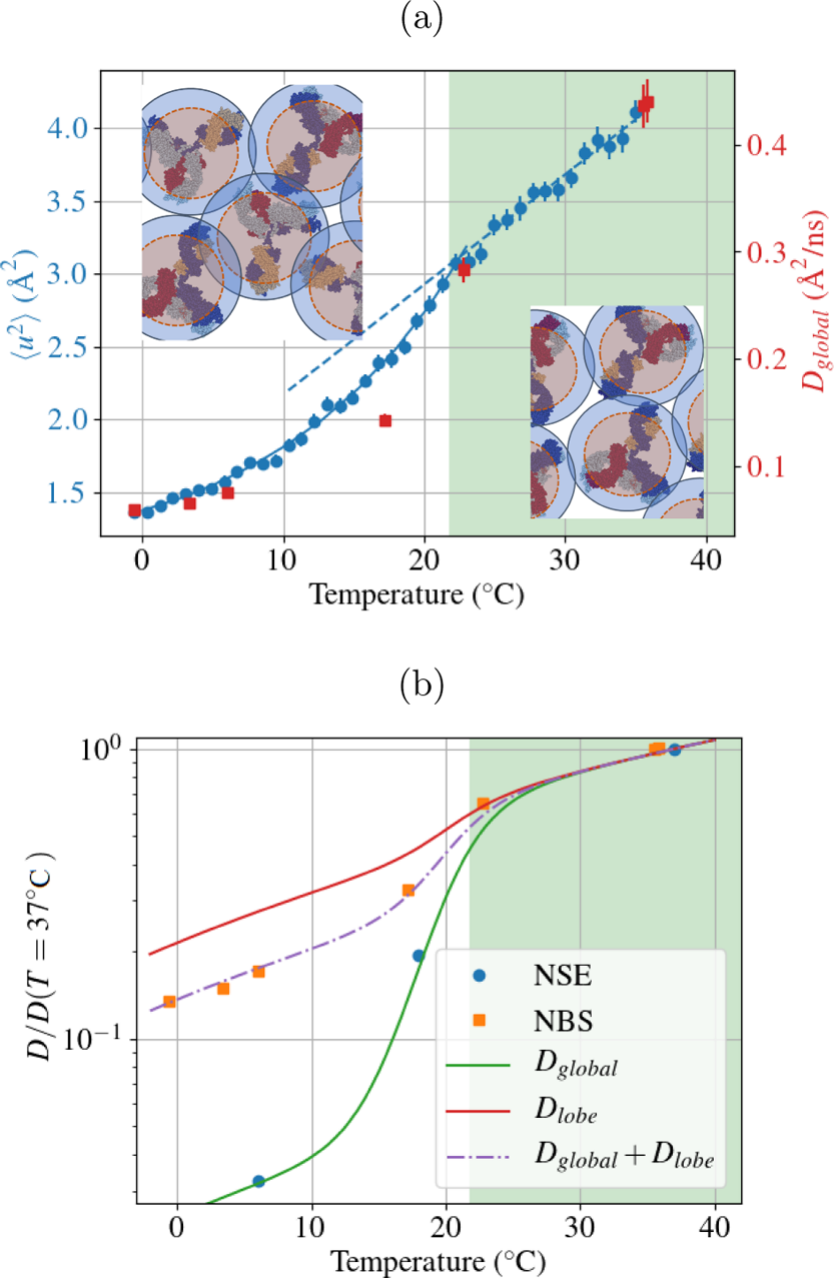
(a) In blue, the apparent mean square displacement (MSD) ⟨*u*^2^⟩ defined by eq 3, as a function of quench temperature is shown. The values were obtained by binning in a temperature range of 0.9 °C. In red, we show the global diffusion coefficient obtained with NBS on a *q* range of 5.8–18.2 nm^–1^. The green shaded area indicates the temperature range in which the solution is stable as a single phase, the dashed line is a linear fit of the points in the one-phase region, and the solid line is a guide to the eye. (b) Diffusion coefficient calculated with the two different techniques. The diffusion coefficient for NSE was calculated, averaging the diffusion coefficient at high *q* (in the range 1.3 nm^–1^ < *q* < 1.75 nm^–1^). We note that the same trend was seen for another sample for which the parent solution had a concentration of 8% PEG. The solid or dashed lines correspond to the fits according to eqs 6–8.

From the NSE data, an effective diffusion coefficient was calculated for the different measured temperatures and *q* values (see inset of Figure 3). Given the almost flat signature as a function of *q*, we averaged the diffusion coefficients at each temperature. The average value of *D* changes drastically in the temperature range measured, with a decrease factor of about 30 (blue circles in Figure 4b). Thus, the relative movements of proteins on the nearest-neighbor scale are strongly reduced, which can be intuitively understood by the proximity of other Y-shaped protein molecules.

To understand when the proximity of other proteins plays a role, we calculated the protein overlap concentration *c*_overlap_ at which the protein lobes start to reach into another protein. To do so, we have to understand at which concentration the average distance between two proteins is equal to their outer radius, for all the concentrations above this value the lobes of two proteins can closely interact. We used *R*_outer_ = 7.7 nm as the value of the outer radius, which is the distance between the center of the protein and the most distant point on a lobe of the antibody, averaged over the three lobes that constitute an antibody. We based our calculation on the PDB structure 1HZH, which was used in the past to describe γ-globulin.^31^ If we assume a random close packing of the particles, hence a volume fraction of ϕ_cp_ = 0.64, the number density of the proteins is given by *n* = ϕ_cp_/*V*_p_, with *V*_p_ = 4/3π*R*_outer_^3^. The protein overlap concentration is then given by
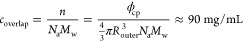
5

Considering that the protein concentration of the parent solution is 100 mg/mL, the concentration of the investigated sample is well above *c*_overlap_ even in the one-phase region; hence, the proteins’ outer radius overlaps at higher concentrations, as represented in the sketch in the right-hand side inset of Figure 4a.

For low temperatures, where the system consists of two phases, the concentration of the dense phase is much higher than *c*_overlap_, and at this point, the proximity of other Y-shaped molecules might extensively slow down the motion of the entire antibody, even if the movement of the lobes could be still present. This observation is consistent with the kinetic slowdown of the phase transition observed on macroscopic length scales, where the growth of the domain size decreases until almost ceasing (Figure S2 of the Supporting Information),^32,46−48^ for which a strong difference in the mobility of dense and dilute phases is expected.

For the same temperature range and sample conditions, the self-diffusion coefficient obtained from the NBS data shows a less severe decrease by a factor of around 5 (red squares in Figure 4a). In the one-phase region (green shaded area), the diffusion coefficient is proportional to the temperature divided by the viscosity of D_2_O, as expected from the Stokes–Einstein equation. Once the phase separation region is reached, a stronger decrease of the diffusion coefficient becomes visible.

The same decrease is observed for the apparent MSD ⟨*u*^2^⟩ from the FWS analysis as a measure of atomic displacements on timescales of few nanoseconds (Figure 4a). Importantly, as these results are recorded during the cooling ramp, we obtain a more continuous profile, which clearly changes from the higher temperature trend once phase separation sets in. It is interesting to note that the MSD and the equilibrium global diffusion coefficient show a similar behavior as a function of temperature, indicating that the system is not aging strongly, as it would be expected in gel and glass phases.

Combining the three data sets from NSE, prethermalized NBS, and FWS–NBS during cooling, we observe significant changes of the diffusive dynamics upon entering in the phase-separated regime. Intuitively, this effect can be understood due to the crowded environment in the dense phase after LLPS. In fact, the dense phase is the majority phase for this initial protein concentration, estimated to have a volume of around 70% of the overall solution. Due to the higher concentration, the proteins in the dense phase clearly dominate the scattering signal (Figure S3a in the Supporting Information). More information on this estimation can be found in section II of the Supporting Information.

### 3.2 Intramolecular Dynamics

The significant additional decrease of the diffusion coefficient from NSE compared to NBS by a factor of more than 5 requires further discussion. First, while the NBS signal is determined by the incoherent neutron scattering, the NSE signal is dominated by coherent scattering, meaning that structural features, that is, spatial correlations, could modify the observed NSE relaxation rate. This effect, the so-called de-Gennes narrowing,^49^ implies that *D*(*q*) follows the inverse of the structure factor *S*(*q*). However, no strong *q* dependence can be observed in the relaxation rate in Figure 3, and de-Gennes narrowing can thus not explain the additional decrease. This is also supported by the small angle neutron scattering (SANS) profiles which do not show a strong correlation peak and, in particular, no significant increase when changing from the homogeneous into the phase-separated and arrested states (for details, see Supporting Information in section I). Second, the different *q* ranges probed by NSE and NBS imply that different motions are probed. In particular, NBS is more sensitive to smaller real-space length scales and thus to intramolecular dynamics of the antibodies, such as lobe motions and local internal motions.

A potential explanation could thus be the different relative contributions of lobe diffusion and self-diffusion of the entire antibody at low and high temperatures, both of which contribute to the experimentally probed displacement in NBS (for a more quantitative estimation, see the end of this paragraph). At higher temperatures, the displacement is dominated by the diffusion of the entire molecule, as indicated by consistent modeling for PEG-free gamma-globulin solutions.^24^ When lowering the temperature to reach the phase-separated regime, the global diffusion is significantly reduced, as observed by NSE. In this situation, the contribution from the lobe diffusion might become more relevant. This can be explained by the fact that the lobes have more motional freedom left, as given their smaller size the effect of the confinement due to the neighbor cage is reduced.

**Figure 5:**
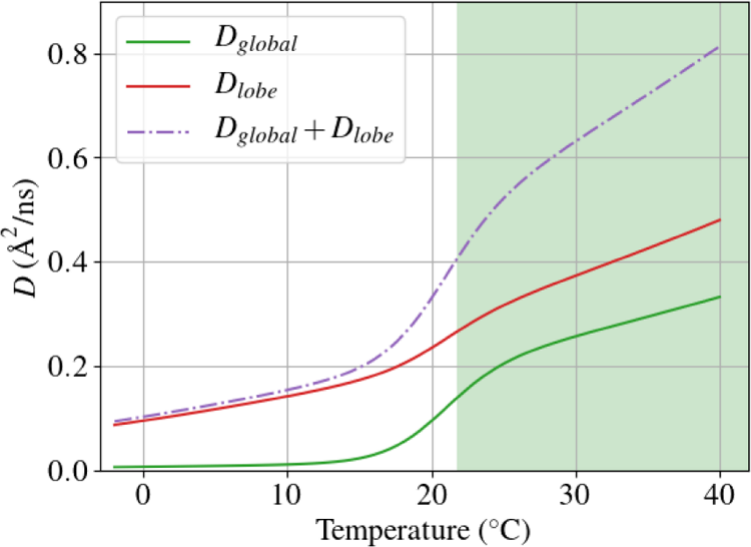
Diffusion coefficients from eqs 7 and 8. The green shaded area corresponds to the one-phase region.

To verify this interpretation, we model the measured short-time diffusion *D*_exp_ as the sum of two contributions, one from the diffusion of the lobes and the second one from the diffusion of the entire antibody
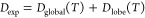
6
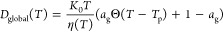
7
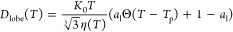
8Here, we use the approximation that the movement of the lobe is not confined on the timescales of nanoseconds probed by NBS. Each of the diffusion coefficient contains not only a temperature dependence which includes temperature and viscosity, as in the Stokes–Einstein equation, but also a step function Θ(*T* – *T*_p_), which models the decrease of the mobility and the possible change in the hydrodynamic radius due to the change in concentration (in the phase-separated region, the signal from the dense phase is predominant), as indicated in eqs 7 and 8. *T*_p_ indicates the temperature at which the phase separation starts, which was set to 20.5 °C. The additional factor  in eq 8 accounts for the difference in the effective radius of an individual lobe with respect to the overall protein. The scalar values *a*_g_ and *a*_l_ express the degree of reduction of the diffusion coefficient. The best agreement of the model with the experimental data can be found for *a*_l_ = 0.3 and *a*_g_ = 0.92 (Figure 4b). These values indicate that *D*_lobe_ decreases of 30% due to the phase separation, and *D*_global_ of 92%, which supports the interpretation that the lobe diffusion is less affected by the crowding of the solution. The values of the diffusion coefficient without the normalization evidence the consequence of the different behaviors of the global and the lobe diffusion coefficients (Figure 5): while at high temperature, they both contribute to the diffusion, at low temperatures, the global diffusion coefficient becomes negligible. As suggested in the beginning of the section, the movement of the whole molecule might be hindered by other molecules, while the small size of the lobe can allow more possibility of movement in local voids, having as a result a decrease of only 30%. In conclusion, the model provides a conclusive picture consistent with the experimental data (Figure 4b).

### 3.3 Geometrical Confinement of Local Dynamics of Protein Residues

As a measure of the local dynamics and flexibility, we analyzed the elastic incoherent structure factor *A*_0_(*q*) (EISF), which provides an estimation of the dynamical confinement of the faster local dynamics (Figure S6 of the Supporting Information). The movements associated with the internal dynamics are very fast on timescales of few to hundreds of picoseconds and do not correspond to the motion of the lobes but rather to the movements of the residues. Following a previous work,^24^ the value of *R* was fixed to , that is, the average distance of the hydrogen atoms in methyl groups (−CH_3_). In this interpretation, the atoms jump between three sites at an angular distance of 120°.

**Figure 6:**
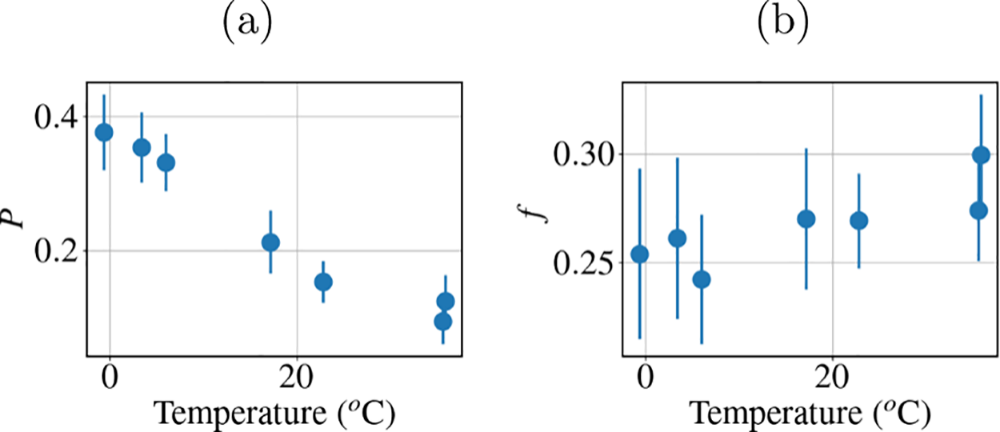
(a) Fraction *p* of fixed hydrogen atoms as a function of temperature. (b) Prefactor *f* from eq 2 as a function of temperature.

The value of the fraction *p* of fixed hydrogen atoms decreases from ∼0.4 to ∼0.1 with increasing temperature (Figure 6a). This trend is expected considering that the thermal energy decreases at low temperature and that the crowding increases in the phase separation region. The prefactor value of *f*, instead, is almost constant (Figure 6b), which is consistent with the assumption that hydrogen atoms within the methyl groups are not strongly affected by the crowding. The local motions becoming more flexible with increasing temperature is thus associated with larger motions of the side chains and the backbone.

## 4 Concluding Discussion

In conclusion, the macroscopically observable phase separation has a strong impact on diffusion on scales of individual antibodies and below, as reflected *inter alia* by the diffusive MSD. By studying the dynamics of antibodies in the dense phase, we mimic the high-density liquid formulations. We interpret the results as an interesting interplay of localized internal motions, diffusion of lobes, and diffusion of the entire molecule. The apparent diffusion coefficients obtained with NSE and NBS spectroscopy show a different temperature dependence, with the first one having a very sharp decrease and the latter a more shallow one. This difference is explained with a simple model, taking into account the diffusion of lobes and the entire antibody. With this model, we show that while at high temperatures the diffusion seems to be controlled by both global and lobe diffusion, at low temperatures, the global diffusion is negligible, and the lobe diffusion dominates the signal. The implication for HCLFs is very relevant, since our findings imply that the dynamics, which is a precondition of the biological function of the protein, is preserved even under highly crowded conditions.

## Supporting Information Available

Characterization of the sample, such as SANS curves and the estimation of the volumes of dense and dilute phases; fit parameters; model used; and techniques employed for the investigation (PDF)
