# Supplementary material for: Molecular Flexibility of Antibodies Preserved Even in the Dense Phase after Macroscopic Phase Separation
Source: Mol Pharm. 2021 Oct 12;18(11):4162–9. doi: 10.1021/acs.molpharmaceut.1c00555 (PMC8564753; doi:10.1021/acs.molpharmaceut.1c00555)
Supplement: Supplementary file 1 — mp1c00555_si_001.pdf [file mp1c00555_si_001.pdf]

Supporting information: Molecular flexibility of antibodies preserved even in dense phase after macroscopic phase separation

Anita Girelli,[1] Christian Beck,[1,2] Famke Bäuerle,[1] Olga Matsarskaia,[2] Ralph Maier,[1] Fajun Zhang,[1] Baohu Wu,[3] Christian Lang,[3] Orsolya Czakkel,[2] Tilo Seydel,[2] Frank Schreiber,[1,*] and Felix Roosen-Runge[4,†]

[1]*Institut für Angewandte Physik, Universität Tübingen, Auf der Morgenstelle 10, 72076 Tübingen, Germany*
[2]*Institut Laue-Langevin, 71 avenue des Martyrs, 38042 Grenoble, France*
[3]*Jülich Centre for Neutron Science JCNS, Heinz Maier-Leibnitz Zentrum (MLZ), Lichtenbergstraße 1,85748 Garching,Germany*
[4]*Department of Biomedical Sciences and Biofilms-Research Center for Biointerfaces (BRCB), Malmö University, Sweden*

(Dated: October 9, 2021)

CONTENTS

I. Small angle scattering 2

II. Estimation of the contribution of dense and dilute phase 3
   A. Experimental procedure to obtain the single phases. 3

III. Fit parameters NBS 3
   A. q-wise fits of the full spectra 3
   B. Global fit of the full spectra 4
   C. Fit of the Elastic Incoherent Structure Factor 4
   D. Fit of the Fixed Window Scans 5

IV. Model lobe and global dynamics contribution 5

V. Quasi-elastic neutron scattering: principles and interpretation 6

References 7

* frank.schreiber@uni-tuebingen.de
† felix.roosen-runge@mau.se

I. SMALL ANGLE SCATTERING

The data shown in this section were collected at the beamlines KWS 2 and KWS 3 at the Forschungs-Neutronenquelle Heinz Maier-Leibnitz (FRM II) in Garching, Germany.

The system was measured at two ranges of the scattering vector ($q = \frac{4\pi}{\lambda}\sin(\theta)$ with 2θ the scattering angle), the first one corresponding to the protein size (Fig. 1 from 0.1 nm^{-1} to 5 nm^{-1}) and the second one corresponding to the domain size (Fig. 2 from 0.002 nm^{-1} to 0.02 nm^{-1}).

In a q range corresponding to the size of the domains the sample was monitored right after the temperature quench by performing consecutive measurements of 1 min exposure each. At 18 °C the intensity changes for the whole measurement time (for a total of more than 30 min), while at 6 °C, instead, a peak forms in the first several minutes, grows until roughly 10 min, after which the growth is strongly reduced (Fig. 2). Importantly at 18 °C the intensity does not show a clear spinodal peak due to the limited time resolution of 1 min,in which the intensity is changing giving as a result the very broad peak visible in Fig. 2b. Since at 6 ° C the intensity change is much slower this effect is less pronounced, having as a result a much more defined peak.

Fig. 1 shows SANS profiles at 37 °C,18 °C and 6 °C. To understand when the structure factor plays a role the intensity was normalised by the intensity at 37 °C. The structure factor in the single-phase is supposed to be very different from the one in the phase separation region, hence if the normalised intensity is constant at 1, this indicates that the q range considered is dominated by the form factor and the structure factor is constant at 1. This is the case for q values larger than 1.4 nm^{-1}.

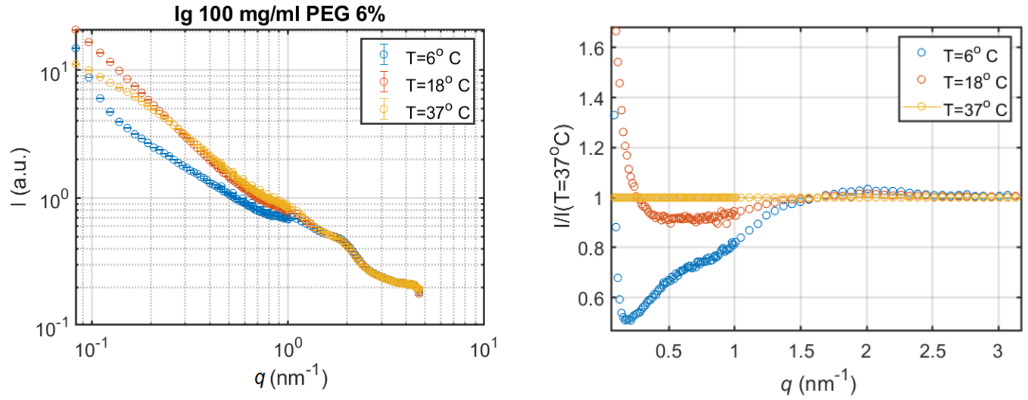

FIG. 1: a) SANS curves measured after equilibration at the temperatures indicated in the legend. b) SANS intensity at 18 °C and 6 °C normalised by the intensity at 37 °C.

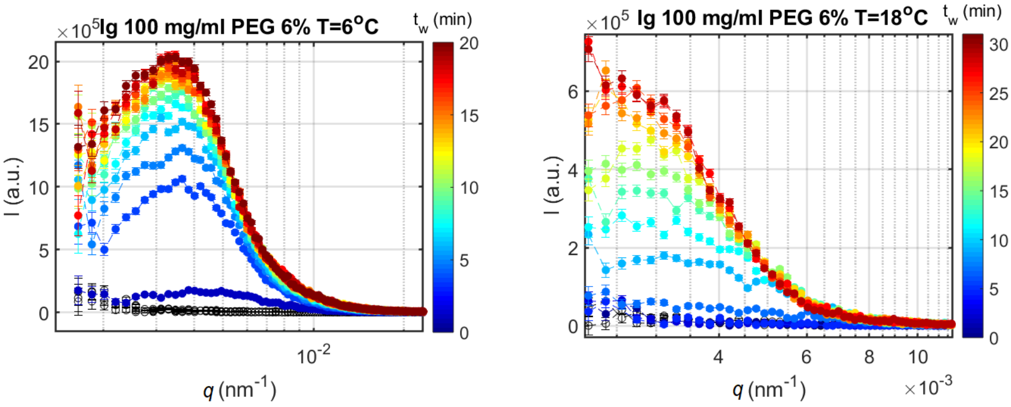

FIG. 2: VSANS curves measured right after the temperature quench from 37 °C to 6 °C (a) and to 18 °C (b). The color corresponds to the waiting time t_w after quench and indicated by the color bar.

II. ESTIMATION OF THE CONTRIBUTION OF DENSE AND DILUTE PHASE

To understand the contribution of the dense and dilute phases to the overall signal of the structure factor measured at IN16b, the single phases formed at 6°C were measured. The experimental procedure to obtain this single phases can be found below. The corresponding dynamics structure factor curves are shown in Figure 3a.

Choosing appropriate weighting factors, the weighted sum of the two signals, $S_{sum} = 0.7S_{dense} + 0.3S_{dilute}$, overlaps with the signal of the sample investigated in this work at 6 °C, which gives us a rough estimate of the percentage of dense and dilute phase in the sample.

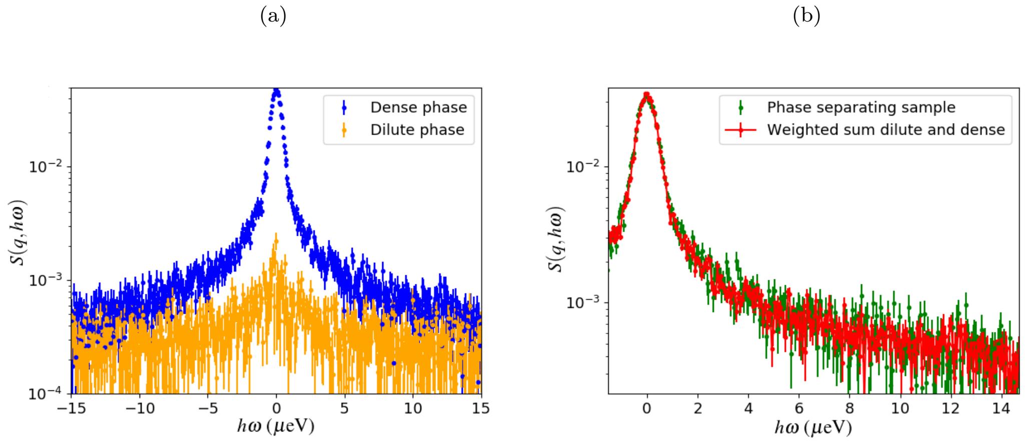

FIG. 3: a) The structure factor from the dilute and dense phase at 6 °C at $q = 1.2\text{Å}^{-1}$. b) The weighted sum ($S_{sum} = 0.7S_{dense} + 0.3S_{dilute}$) of the two signals shown in (a), and their parent solution after a quench to 6 °C.

A. Experimental procedure to obtain the single phases.

To obtain the dense phase the sample was centrifuged for 24 h at 6 °C. After this process two phases were present. The dilute phase was transparent, but the dense phase was highly turbid. This indicates that some part of the dilute phase was still present in the dense phase in the form of small droplets. Given the very low concentration of the dilute phase and the small amount this contribution was considered negligible.

For the extraction of the dilute phase a different method was followed since the amount of dilute phase after the procedure was small, as expected from the lever rule for a binary mixture with an initial concentration close to the dense phase branch. After the parent solution of the investigated sample was equilibrated at 21°C the dilute phase was extracted and then equilibrated at 6 °C. Again the solution separated 2 in liquid phases, and the less concentrated one was used as the dilute phase of the sample at 6 °C.

III. FIT PARAMETERS NBS

A. q-wise fits of the full spectra

Before performing a global fit fits of the curves $S(q, h\omega)$ at fixed q were performed to check the validity of the assumption of Fickian diffusion for the apparent global diffusion and the diffusion of PEG and jump diffusion for the internal dynamics of the protein.

While it is possible to see that the trend of the points is following the said type of dynamics, the fit is unstable due to the high number of parameters, especially for small q values. For this reason a global fit was performed.

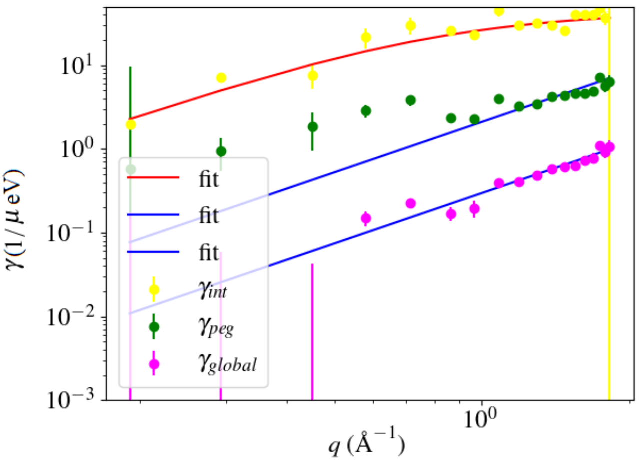

FIG. 4: The decorrelation times extracted from the q-wise fit. The blue solid line correspond to a fit of a fickian diffusion, and the red one to a jump diffusion type of fit.

B. Global fit of the full spectra

In this section the fit parameters of the NBS data not included in the main text are shown, in particular the diffusion coefficients of PEG and the internal dynamics from the global fit of the whole spectra (Fig. 5). The fits are called global because it is three dimensional since it depends on both q and *omega*.

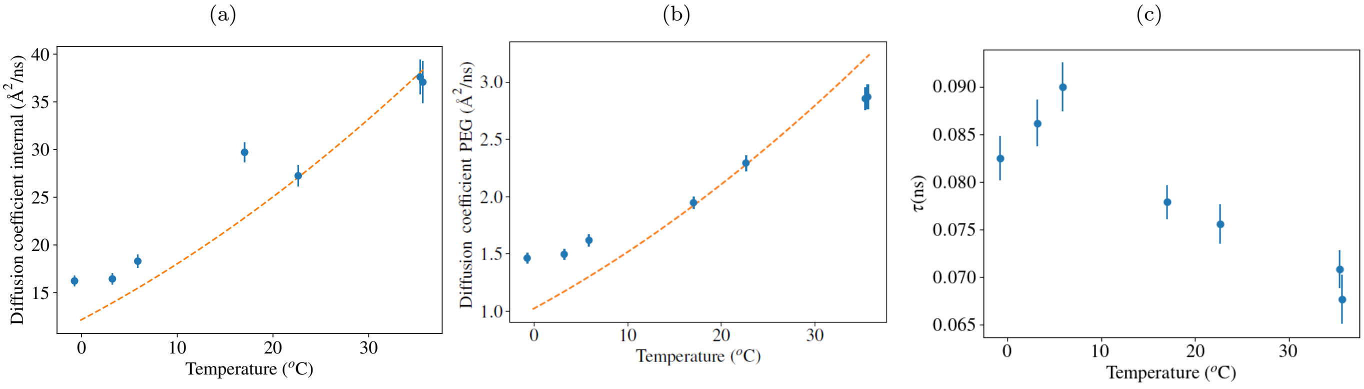

FIG. 5: Diffusion coefficients of the internal dynamics of the protein (a) and the diffusion coefficient of the polymer (b) as a function of temperature. The dashed line shows the temperature dependence that is expected from the Stokes-Einstein equation, i.e. $D \propto \frac{T}{\eta(T)}$. c) The residence time τ of the jump diffusion as a function of temperature.

C. Fit of the Elastic Incoherent Structure Factor

The parameter $A_0(q)$ was fitted with equation 1. The value of R was fixed to 1.715 Å, since it is the average distance of the hydrogen atoms in the methyl groups (-CH_3). The parameters obtained are shown in Fig. 6

$$A_0(q) = f \exp(-R^2q^2) + P \tag{1}$$

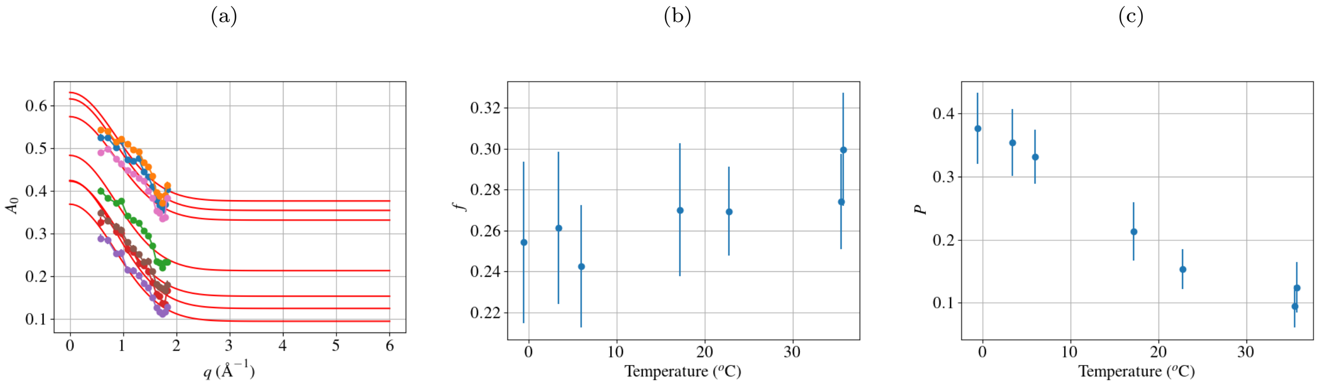

FIG. 6: a) The parameter $A_0(q)$ obtained from the global fit of the NBS full spectra measured at different temperatures as a function of q, fitted according with equation 1. The fit parameters f (b) and P (c) as a function of temperature.

D. Fit of the Fixed Window Scans

The data points were fitted with equation 2 as described in the main text.

$$\log(S(q,\omega=0)) = I_0 - \frac{1}{3}q^2\langle u^2\rangle + bq^4 \tag{2}$$

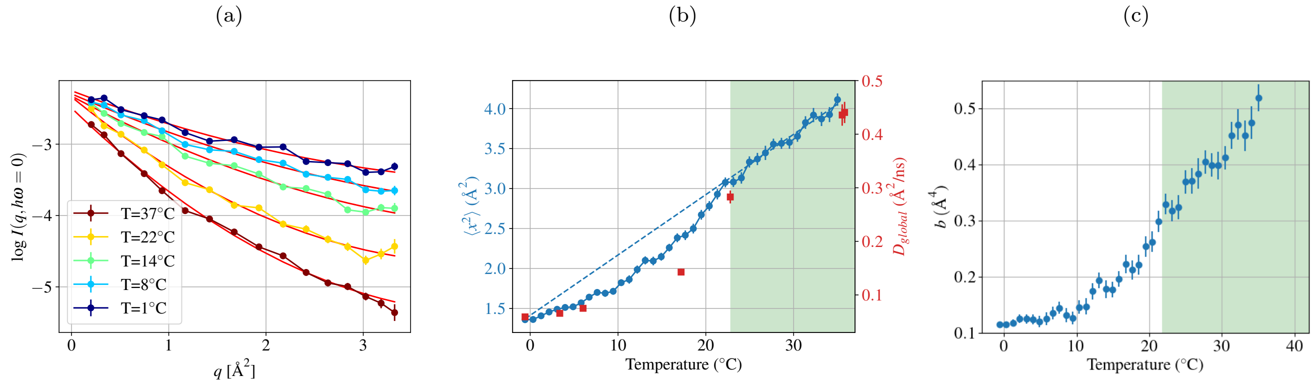

FIG. 7: a) The fit of the fixed windows scan intensities at different temperatures as indicated in the legend. The red curves correspond to the fit according to equation 2. The fit parameters $\langle x^2\rangle$ and b are displayed as a function of temperature in (b) and (c).

IV. MODEL LOBE AND GLOBAL DYNAMICS CONTRIBUTION

The analytical function that describes the dependency of the diffusion coefficients on the temperature are illustrated in equations 3 and 4. The value of K_0 was chosen so that D_{global} has a value of 1 Å^2/ns at 37 °C. The pre-factor $\frac{1}{3}^{\frac{1}{3}}$ was added to the lobe diffusion to take into account the different effective radii, more specifically the volume of a single lobe was approximated to be $\frac{1}{3}$ of the volume of the protein. a_g and a_l have a value of 0.3 and 0.92, respectively. The parameters were chosen in order to best fit the experimental data.

$$D_{global}(T) = \frac{K_0 T}{\eta(T)}\left(a_g\Theta(T-T_p) + 1 - a_g\right) \tag{3}$$

$$D_{lobe}(T) = \frac{K_0 T}{\frac{1}{3}^{\frac{1}{3}} \eta(T)} \left(a_l \Theta(T - T_p) + 1 - a_l \right) \tag{4}$$

The function $S(T - T_P)$ defined in equation 5, in which T_p is the temperature at which the phase separation starts, and k is a constant that sets how steep the sigmoidal function is. Its value was set to 2.1 K.

$$\Theta(T - T_P) = \frac{1}{1 + a \exp - \frac{T - T_p}{k}} \tag{5}$$

The diffusion coefficients dependency on temperature can be seen in Fig. 8.

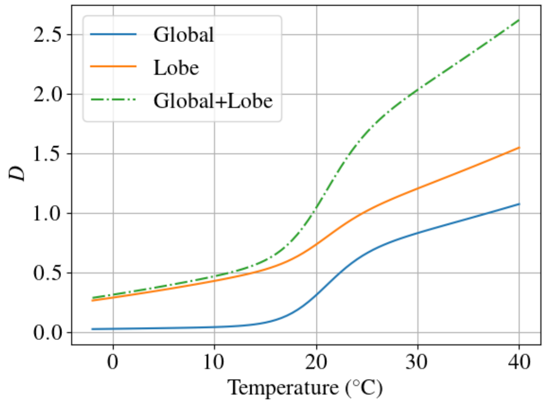

FIG. 8: The diffusion coefficients dependency on temperature.

V. QUASI-ELASTIC NEUTRON SCATTERING: PRINCIPLES AND INTERPRETATION

During the scattering process of a neutron in a sample, the neutron exchanges both momentum and energy with the sample. While the change of momentum is linked to a change of direction, and exploited in static scattering techniques, the change of energy provides access to motions in the sample on time scales ranging from picoseconds to many nanoseconds [2, 3].

The measurement of the neutron energy can be realized with different methods. Neutron backscattering spectroscopy uses crystal reflection at a monochromator and analyzer to obtain a well-defined neutron energy, and then uses Doppler shifting the monochromator to realize different energy shifts. Neutron time-of-flight spectroscopy simply measures the flight time of neutrons as a proxy of their kinetic energy [2]. Neutron spin echo spectroscopy uses a more advanced way to measure flight time, employing the precession of spins in well-adjusted magnetic fields as a very precise stop watch [4].

Importantly, the basic observables from quasi-elastic neutron scattering are directly linked to spatio-temporal correlation functions, i.e. well-defined statistical quantities encoding the dynamical processes in the sample.

In the case of antibody solutions, diffusion processes such as translational diffusion or diffusive motions of lobes and protein sidechains dominate the dynamics. As outlined in the main manuscript, the experimental scattering functions directly deliver information on the relaxation rates on specific length scales. Beside the absolute value of relaxation rates, the change of relaxation rates depending on the length scale provide important insights into the nature of the dynamics, such as dynamical confinement or correlated motions.

In this context, it is important to stress that different techniques such as neutron backscattering and neutron spin echo access slightly different observables, and dynamical contributions to the relaxation are weighted in different ways. On the one hand, neutron backscattering at larger q values is mainly determined by the self-motion of hydrogens, which comprise a combination of the diffusion of the entire molecule and internal fluctuations. On the other hand, neutron spin echo at lower q is dominated by the coherent scattering, representing collective and concerted motions across the molecule. Importantly, the collective diffusion approaches self-diffusion in the high-q limit, and neutron

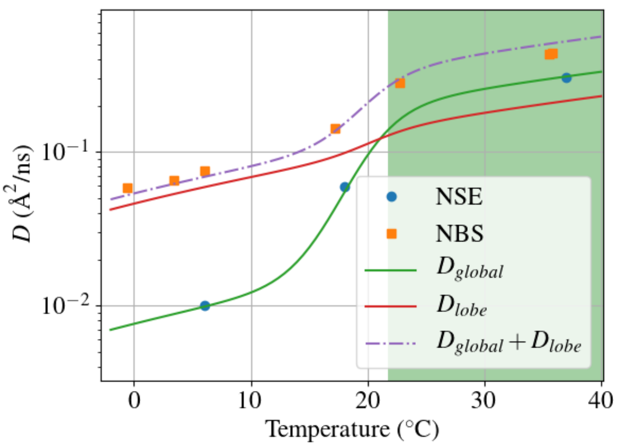

FIG. 9: Diffusion coefficient from neutron spin echo and neutron backscattering on an absolute scale
.

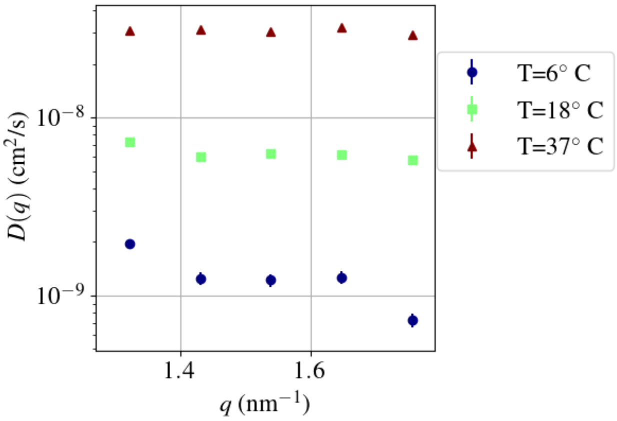

FIG. 10: The diffusion coefficient obtained from the NSE data. Different colors correspond to different temperatures as indicated in the legend.

backscattering and neutron spin echo thus deliver information with comparable content. However, slight differences can persists due to a different weighting of dynamical contributions. As an example, we show the diffusion coefficients obtained from neutron spin echo and neutron backscattering on an absolute scale in Fig. 9.

In figure 10 the values of the diffusion coefficient obtained from the analysis of the NSE data are shown in cm^2/s for easier comparison with the literature.

---

[1] S. Da Vela, F. Roosen-Runge, M. W. A. Skoda, R. M. J. Jacobs, T. Seydel, H. Frielinghaus, M. Sztucki, R. Schweins, F. Zhang, and F. Schreiber, The Journal of Physical Chemistry B **121**, 5759 (2017).

[2] M. Bée, *Quasielastic neutron scattering* (Adam Hilger, Bristol, 1988).

[3] M. Grimaldo, F. Roosen-Runge, F. Zhang, F. Schreiber, and T. Seydel, Quarterly Reviews of Biophysics **52** (2019).

[4] F. Mezei, *The principles of neutron spin echo* (Springer, 1980).
